# COVID-19 Dysautonomia

**DOI:** 10.3389/fneur.2021.624968

**Published:** 2021-04-13

**Authors:** Brent P. Goodman, Julie A. Khoury, Janis E. Blair, Marie F. Grill

**Affiliations:** ^1^Department of Neurology, Mayo Clinic, Scottsdale, AZ, United States; ^2^Infectious Disease, Mayo Clinic, Scottsdale, AZ, United States

**Keywords:** postural tachycardia syndrome, COVID-19, autonomic neuropathy, orthostatic hypotension, POTS, dysautonomia

## Abstract

**Objective:** To report a case series of dysautonomia associated with COVID-19 infection.

**Methods:** This is a retrospective review of patients evaluated in the autonomic clinic at our institution with suspected signs and symptoms of dysautonomia who underwent formal evaluation, including autonomic testing.

**Results:** Six patients were identified with signs and symptoms suggestive of dysautonomia who underwent autonomic testing. All patients had symptoms typical of COVID-19 infection, though none were hospitalized for these or other symptoms. All patients reported symptoms of postural lightheadedness and near-syncope, fatigue, and activity intolerance. Five patients reported the onset of autonomic symptoms concomitant with other COVID-19 symptoms, with the other patient reporting symptom onset 6 weeks following initial COVID-19 symptoms. Autonomic testing demonstrated an excessive postural tachycardia in 4 patients, a hypertensive response with head-up tilt in 3 patients, orthostatic hypotension in 1 patient, and sudomotor impairment in 1 of the patients with excessive postural tachycardia.

**Conclusions:** We present clinical features and results of autonomic testing in 6 patients with a history COVID-19 infection. While all patients reported typical features of orthostatic intolerance, fatigue, and activity intolerance, the results of autonomic testing were heterogenous, with orthostatic hypotension in 1 patient, excessive postural tachycardia typical of postural tachycardia syndrome in 4 patients, and postural hypertension in 3 patients.

## Introduction

The clinical spectrum and consequences of the worldwide pandemic due to coronavirus disease 2019 (COVID-19) are now being collated. Neurological manifestations resulting from infection by severe respiratory syndrome coronavirus 2 (SARS-CoV-2) in hospitalized patients have been a focus of early studies (1–3). There is at present even less understanding of the potential medical and neurological sequelae in non-hospitalized COVID-19 patients, and particularly, individuals with persistent symptoms following SARS-CoV-2 infection.

Herein, we report the clinical features and results of formal autonomic testing in a cohort of non-hospitalized COVID-19 patients with persistent, disabling symptoms due to dysautonomia. That dysautonomia should occur in conjunction with or following COVID-19 should be expected, given a reported prevalence of dysautonomia in 50% of severe acute respiratory syndrome (SARS) patients studied in the 2002 SARS epidemic (4), and that the most prevalent, primary autonomic disorder, postural tachycardia syndrome (POTS), is commonly associated with antecedent or concomitant infection.

## Methods

This retrospective review was approved by our institutional review board. All but 1 patient included in this study were diagnosed with COVID-19 via a nasal swab PCR SARS-CoV-2 assay; the remaining patient tested positive for IgM and IgM SARS-CoV2 antibodies. All patients underwent a thorough medical history and examination, were identified to have potential autonomic signs and symptoms by one of the authors, and had laboratory testing to exclude mimickers of autonomic dysfunction (5). Autonomic testing was conducted in all patients through techniques established in Mayo Autonomic Laboratories. This included an assessment of sweating at 4 sites in the extremities through quantitative sudomotor axon reflex screen, evaluation of heart rate variability (cardiovagal function) with deep breath and the Valsalva maneuver, and tilt-table testing (TTT), performed with continuous monitoring of heart rate and blood pressure (6, 7). These tests allow for an assessment of postganglionic sympathetic sudomotor, cardiovagal, and cardiovascular adrenergic function. Adrenergic testing was performed by assessing blood pressure response to Valsalva maneuver and during head-up tilt utilizing continuous, beat-to-beat heart rate and blood pressure monitoring (6). Analysis of blood pressure responses to the Valsalva maneuver was analyzed to assess baroreflex function, through analysis of phase II early, phase II late, and phase IV responses. Analysis of heart rate and blood pressure responses to head-up tilt, provided additional assessment of adrenergic tone, utilizing 70 degree tilt-table testing for 10 min. A sustained heart rate increment in excess of 30 beats-per-minute in the absence of orthostatic hypotension, was considered abnormal as per usual protocol (5). Orthostatic hypotension was identified with a sustained decrease in systolic blood pressure of 20 mm Hg or a decrease in diastolic blood pressure of 10 mm Hg. Tests of cardiovagal function were performed by evaluating heart rate responses to deep breathing and the Valsalva maneuver (6), with results based upon normal values established in Mayo Clinic Autonomic Laboratories. Medications with the potential to impact autonomic testing were reviewed prior to and at the time of autonomic testing, and those with the potential to impact testing were discontinued.

## Results

A total of 6 patients were identified with COVID-19 infection who underwent formal autonomic evaluation in our clinic and autonomic laboratories (see Table 1). Patients ranged in age from 22 to 66, and 4 of the 6 patients were female. None of the patients were hospitalized for COVID-19 infectious symptoms, and none of the patients were diagnosed with pneumonia. Chest imaging with either chest XR or computed tomography was performed in 3 patients and was unremarkable. A complete blood count and electrolytes were performed in all patients and was negative. All patients had at least some symptoms typical of SARS-CoV2 at disease onset, including fever in 5, cough in 4, and shortness of breath in all but 1 patient. None of the patients reported a history of antecedent autoimmune disease, aside from 1 patient with eczema, and none of the patients reported an antecedent history of a chronic medical condition with the potential to result in deconditioning or of autonomic dysfunction.

**Table 1:** Patient demographics and features—COVID-19 dysautonomia.

**Age**	**Gender**	**Autonomic symptoms**	**Other symptoms**	**Autonomic testing results**
30	Female	Postural LH, Syncope, Near-syncope, Palpitations Chest Pain	Headache, SOB, dysesthesias Dysgeusia, ansomia	Postural Tachycardia & HTN
22	Female	Postural LH, Syncope, Near-syncope, Palpitations	Headache, SOB, anosmia	Postural Tachycardia
45	Male	Postural LH, Palpitations	SOB, extremity dysesthesias	Postural Tachycardia
49	Female	Postural LH, Near-syncope Palpitations, Chest Pain	Headache, SOB	Postural Tachycardia & HTN Decreased sweating foot
66	Male	Postural LH, Near-syncope	Vestibular symptoms	Orthostatic Hypotension Cardiovagal impairment
55	Female	Postural LH, Near-syncope Chest Pain	Headache, SOB, dysgeusia, anosmia	Postural Hypertension Cardiovagal impairment Postural Hyperventilation

*Clinical Features and Autonomic Findings in 6 COVID-19 patients with Dysautonomia. LH, lightheadedness; SOB, shortness of breath; HTN, hypertension*.

All patients reported a history of postural lightheadedness and near-syncope, and heart racing and palpitations were reported by 5 of 6 patients. All patients reported persistent fatigue and activity intolerance. Symptoms suggestive of autonomic dysfunction began with other COVID-19-related symptoms in 5 of 6 patients, and in the remaining patient began 6 weeks following the onset of otherwise typical—COVID-19-related symptoms. Intermittent chest pain was reported by 3 patients, loss of sense or smell was reported by 3 patients, and dysesthesias were reported by 2 patients. None of the patients in this cohort reported new onset gastrointestinal or genitourinary symptoms. A new onset and persistent headache was reported by 4 patients and was reported by 2 of the patients to be distinct from their typical migraine headaches experienced prior to COVID-19 infection. Detailed medical and neurological examination was normal in all patients.

**Figure 1:**
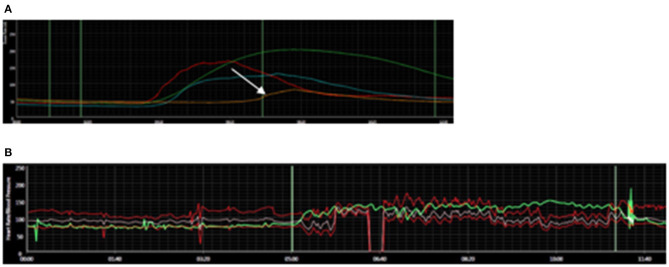
Autonomic Findings in COVID-19 Dysautonomia patient. **(A)** Reduced sweating on quantitative sudomotor axon reflex testing in the foot (arrow); normal elsewhere. **(B)** Abnormal, excessive increase in heart rate (61 beats per minute) with head-up tilt. Heart rate is depicted in green. Blood pressure in red.

Autonomic testing was abnormal in all patients tested, and the findings were notably heterogenous (see Table 1). None of the patients were on medications with the potential to impact findings at the time of autonomic testing. Mean time from COVID-19 symptom onset to autonomic testing was just under 3 months, ranging from 1 to 4 months (see Table 2). An abnormal heart rate increment was noted with head-up tilt (HUT) in 4 patients, ranging from 33 to 60 beats per minute (bpm) in these patients. An abnormal increase in blood pressure was seen in 3 patients during HUT. Orthostatic hypotension with inadequate heart rate increment was seen in 1 patient, and this patient also demonstrated an abnormal blood pressure response to the Valsalva maneuver. Abnormal sudomotor function was noted in 1 patient, with a reduction in sweating in the foot, and normal sweating elsewhere. This patient also had a 60 bpm increase in heart rate during HUT (see Figure 1). Nerve conduction studies and needle EMG were performed and were normal in the 2 patients reporting dysesthesias.

**Table 2:** Results of autonomic testing.

**Patient**	**Time to testing**	**Sudomotor**	**HR DB**	**Sup HR**	**Sup BP**	**HUT HR max**	**HUT BP mean**	**Max HR increment**
1	4 months	Normal	Normal	68	114/72	101	131/86	33
2	3 months	Normal	Normal	67	114/70	107	108/75	40
3	2.5 months	Normal	Normal	63	128/89	120	127/93	57
4	2 months	Reduced Foot	Normal	82	111/73	143	124/95	61
5	1 months	Normal	Reduced	59	153/71	73	130/65	14
6	4 months	Normal	Reduced	73	131/86	99	151/96	26

*HR refers to heart rate, recorded in beats per minute. DB, deep breathing; Sup, supine; HUT, head-up tilt; BP, blood pressure in mm Hg. Supine heart rate and blood pressure was recorded immediately prior to head-up tilt. Mean blood pressure was calculated using recorded cuff measurements at 3, 5, 7, and 10 min following head-up tilt*.

## Discussion

We present the clinical features and findings on autonomic testing in a cohort of 6 patients with typical symptoms of dysautonomia associated with COVID-19 infection. While patients all reported typical symptoms of orthostatic intolerance, including postural lightheadedness, palpitations, shortness of breath, and near-syncope; the findings on autonomic testing were heterogenous. An excessive postural tachycardia was seen in 4 of 6 patients, orthostatic hypotension and cardiovagal impairment typical of an autonomic neuropathy was present in 1 patient, and postural hypertension and cardiovagal impairment was seen in 1 patient. Notably, autonomic symptoms were present at COVID-19 onset in 5 of the 6 patients in this series, and none of the patients were hospitalized for these or other symptoms.

As of this time, reports of COVID-19-related autonomic dysfunction have been limited. Dizziness was reported as a symptom in 16.8% of 214 hospitalized patients in Wuhan, China (3). Dizziness is not further characterized however in this series, and the study population was limited to patients with severe acute respiratory syndrome. In another large review of 841 hospitalized COVID-19 patients, dysautonomia was reported in 21 (2.5%) patients (1). Dysautonomia was considered mild in 15 of these patients, though further details are not described. A case of postural tachycardia syndrome (POTS) following COVID-19 infection, reported the development of autonomic symptomatology simultaneous with fever, cough, and shortness of breath in a 26-year-old female (8). Autonomic testing in this patient performed over 3 months following symptom onset showed postural hypertension and tachycardia, as seen in 2 of the patients in our cohort. Recurrent, convulsive syncope was reported as an initial manifestation of COVID-19 infection in a 70-year-old female who subsequently developed dyspnea and hypoxia (9). A sympathetic skin response was reportedly abnormal in this patient. While the first case series of 5 patients with Guillain-Barre syndrome in association with COVID-19 reported 0 of 5 patients as having dysautonomia, tachycardia along with alternating hypotension and hypertension was subsequently reported in a 72-year-old male with Guillain-Barré syndrome (10, 11). Another case of autonomic failure was reported in a 20-year-old male, who developed postural dizziness, sweating, constipation, erectile dysfunction, chest pain, and shivering, 3 days after developing a fever and malaise due to COVID-19 infection (12). Examination of this patient revealed a postural decrease in blood pressure, generalized weakness, and areflexia; with electrodiagnostic testing suggesting acute motor axonal neuropathy (AMAN).

Autonomic testing in 4 of 6 patients in our series revealed findings potentially compatible with POTS, including 2 patients with both hypertension and tachycardia on HUT, consistent with a hyperadrenergic POTS subtype. POTS is widely considered to be the most common primary autonomic disorder, and is characterized by a heart rate increment that exceeds 30 BPM in adults, or 40 BPM in individuals < 18 years of age, within 10 min of standing or on TTT (5). It is notable that 40–50% of POTS patients report an antecedent history of infection prior to symptom onset (13, 14). Various types of infections have been reported in association with the development of POTS, including Mycoplasma pneumonia (15), Epstein Barr virus (16, 17), Trypanosoma cruzii (18), and Borrelia burgdorferi (19, 20). While not definitively established to this point, it has been generally assumed that the development of autonomic nervous system impairment in POTS patients is a para- or post-infectious, immune-mediated process. That the autonomic nervous system is vulnerable to such a process is supported by presence of dysautonomia in patients with Guillain-Barré syndrome (21) and in acute autoimmune autonomic neuropathy (22), the autonomic version of Guillain-Barré syndrome.

It is, however unclear, whether dysautonomia associated with COVID-19 results directly from the effects of virus on autonomic pathways, or through para-infectious or post-infectious immune-mediated mechanisms. A recent, comprehensive review providing a detailed summary of the various central nervous system and peripheral nervous system complications of COVID-19 emphasizes this point; with the authors suggesting that even in cases of COVID-19 related encephalitis, the comparative importance of direct viral invasion and the immunologic response to it, is not established (2). The onset of autonomic symptomatology concurrent with the onset of COVID-19 infection in 5 of 6 patients in this series and in the aforementioned case reports would argue against deconditioning as a mechanism for dysautonomia in these patients.

Given the small number of patients in our series and those previously reported, localization to central or peripheral nervous autonomic pathways cannot be determined with certainty. The finding in 1 patient of sudomotor impairment in conjunction with an excessive postural tachycardia can be reasonably assumed to reflect an autonomic neuropathy. The report of dysesthesias in 2 of the patients in our series would suggest involvement of somatic, small fiber nerves, and it would be reasonable to assume that autonomic nerves might also be impaired. As of the time of this writing, skin biopsy to assess epidermal nerve fiber density has not been pursued in any of the patients in our series. The presence of postural hypertension in 3 of the patients in our series and in the previously mentioned case report is suggestive of a hyperadrenergic state (8). Localization to central or peripheral autonomic pathways has not been established in hyperadrenergic POTS; a common condition, that these patients phenotypically most closely resemble.

Though the scope of COVID-19 dysautonomia is at this point unknown, the implications and significance are enormous given the scale of the pandemic. It is at this point unclear whether dysautonomia is more prevalent in COVID-19 than other infections or whether there are any mechanisms that uniquely target vulnerable autonomic pathways. Ideally, prompt recognition of potential autonomic symptomatology and subsequent evaluation would lead to initiation of appropriate treatment and improvement in outcome in those suffering from persistent symptoms following COVID-19 infection. It remains to be determined, whether and to what extent patients with the so-called “long haul syndrome” are suffering from dysautonomia (23). A recent commentary from France may provide some general sense of the scope of persistent symptoms post-COVID, reporting an average of 30 individuals per week with symptoms of fatigue, myalgia, sensation of fever, shortness of breath, chest tightness, tachycardia, headaches, and anxiety (24). The authors of this commentary did not comment on whether postural vital signs were performed in these individuals, and presumably autonomic testing was not performed (despite the authors reporting that symptoms resembled dysautonomia). If abnormal autonomic findings are present in some percentage of “long haul syndrome” patients, it would be reasonable to expect that treatment directed toward dysautonomia might lead to clinical improvement.

This study has a number of limitations, including small study size, retrospective study design, and absence of long-term follow-up. While all patients were evaluated by clinicians with autonomic expertise and underwent formal autonomic testing, formal autonomic questionnaires were not administered to patients in this cohort. Future studies of dysautonomia in COVID-19, particularly those that are interventional or study outcomes, may benefit from administration of COMPASS-31 (25) or other quality of life measures in better understanding disease burdens, functional impact, and prognosis.

## Conclusions

We present clinical and autonomic laboratory findings in a small cohort of patients with dysautonomia associated with COVID-19 infection. Autonomic testing in 4 of these patients revealed findings of an excessive postural tachycardia as is seen in POTS, 1 patient had orthostatic hypotension on tilt-table testing, and 3 patients appeared hyperadrenergic. We recommend further studies to explore the prevalence, pathophysiology, clinical features, and treatment approach in patients with COVID-19-related dysautonomia.

## Data Availability Statement

The raw data supporting the conclusions of this article will be made available by the authors, without undue reservation.

## Ethics Statement

The studies involving human participants were reviewed and approved by Mayo Clinic Institutional Review Board. Written informed consent for participation was not required for this study in accordance with the national legislation and the institutional requirements.

## Author Contributions

All authors contributed to manuscript preparation and critical review.

## Conflict of Interest

The authors declare that the research was conducted in the absence of any commercial or financial relationships that could be construed as a potential conflict of interest.
